# Supplementary material for: At What Age Could Screening for Familial Retinoblastoma Be Discontinued? A Systematic Review
Source: Cancers (Basel). 2021 Apr 17;13(8):1942. doi: 10.3390/cancers13081942 (PMC8072927; doi:10.3390/cancers13081942)
Supplement: Supplementary file 1 [file cancers-13-01942-s001.zip › cancers-1143737-supplementary.pdf]

cancers

MDPI

Commentary

Supplementary Material: At What Age Could Screening for Familial Retinoblastoma be Discontinued? A Systematic Review.

Milo van Hoefen Wijsard [1,*,†], Saskia H. Serné [1,†], René H. Otten [2], Machteld I. Bosscha [1], Charlotte J. Dommering [3], Armida W. Fabius [1] and Annette C. Moll [1]

Literature search strategy

Table S1. PubMed search on Feb 12 2021 (661).

Search	Query	Results
#15	#14 NOT *[611 PMIDs]*	50
#14	#7 OR #9 OR #11 OR #13	661
#13	#5 AND #12	301
#12	"Age Factors"[Mesh:NoExp] OR "Age of Onset"[Mesh] OR "age at detection"[tiab] OR "age at diagnosis"[tiab] OR "age at onset"[tiab] OR "age at presentation"[tiab] OR "age of detection"[tiab] OR "age of diagnosis"[tiab] OR "age of onset"[tiab] OR "age of presentation"[tiab] OR "detection age"[tiab] OR "diagnosis age"[tiab] OR "onset age"[tiab] OR "presentation age"[tiab]	539,778
#11	#5 AND #10	91
#10	"Registries"[Mesh] OR registry[tiab] OR registries[tiab] OR register[tiab] OR registers[tiab]	244,265
#9	#5 AND #8	136
#8	"Early Diagnosis"[Mesh] OR early diagnos*[tiab] OR early detect*[tiab]	188,186
#7	#5 AND #6	239
#6	"Mass Screening"[Mesh] OR screen*[tiab]	834,559
#5	#3 OR #4	3,468
#4	"Retinoblastoma/congenital"[Mesh] OR "Retinoblastoma/genetics"[Mesh]	2,301
#3	#1 AND #2	1,844
#2	familial[tiab] OR hereditary[tiab] OR inheritable[tiab] OR bilateral[tiab]	438,509
#1	"Retinoblastoma"[Mesh] OR Retinoblastoma*[tiab] OR Retinal Neuroblastoma*[tiab] OR Retinal Glioma*[tiab] OR "Retinal	18,066

Glioblastoma"[tiab] OR "Retinal Glioblastomas"[tiab]

Final search highlighted in yellow.

Table S2. Embase.com search on Feb 12 2021 (736).

No.	Query	Results
#15	#14 NOT [656 PUIs]	81
#14	#7 OR #9 OR #11 OR #13	736
#13	#5 AND #12	324
#12	'onset age'/exp OR ((age NEAR/4 detection):ti,ab,kw) OR ((age NEAR/4 diagnosis):ti,ab,kw) OR ((age NEAR/4 onset):ti,ab,kw) OR ((age NEAR/4 presentation):ti,ab,kw)	225804
#11	#5 AND #10	83
#10	'register'/exp OR registry:ti,ab,kw OR registries:ti,ab,kw OR register:ti,ab,kw OR registers:ti,ab,kw	349612
#9	#5 AND #8	167
#8	'early diagnosis'/exp OR 'early diagnos*':ti,ab,kw OR 'early detect*':ti,ab,kw	267776
#7	#5 AND #6	308
#6	'mass screening'/exp OR screen*:ti,ab,kw	1220728
#5	#3 OR #4	2556
#4	'retinoblastoma'/exp/dm_cn	169
#3	#1 AND #2	2466
#2	familial:ti,ab,kw OR hereditary:ti,ab,kw OR inheritable:ti,ab,kw OR bilateral:ti,ab,kw	613579
#1	'retinoblastoma'/exp OR retinoblastoma*:ti,ab,kw OR 'retinal neuroblastoma*':ti,ab,kw OR 'retinal glioma*':ti,ab,kw OR 'retinal glioblastoma*':ti,ab,kw	23402

Final search highlighted in yellow.

Table S3. Ebsco/CINAHL search on Feb 12 2021 (105).

#	Query	Results
S13	S12 NOT [661 PMIDs]	41
S12	S5 OR S7 OR S9 OR S11	105
S11	S3 AND S10	35
S10	(MH "Age Factors") OR (MH "Age of Onset") OR TI ((age N3 detection) OR (age N3 diagnosis) OR (age N3 onset) OR (age N3 presentation)) OR AB ((age N3 detection) OR (age N3 diagnosis) OR (age N3 onset) OR (age N3 presentation)) OR SU ((age N3 detection) OR (age N3 diagnosis) OR (age N3 onset) OR (age N3 presentation))	166,856
S9	S3 AND S8	15
S8	(MH "Registries, Disease") OR TI (“registry” OR “registries” OR “register” OR “registers”) OR AB (“registry” OR “registries” OR “register” OR “registers”) OR SU (“registry” OR “registries” OR “register” OR “registers”)	81,337
S7	S3 AND S6	31
S6	(MH "Early Diagnosis+") OR TI (“early diagnos*” OR “early detect*”) OR AB (“early diagnos*” OR “early detect*”) OR SU (“early diagnos*” OR “early detect*”)	44,682
S5	S3 AND S4	51
S4	(MH "Health Screening+") OR TI (screen*) OR AB (screen*) OR SU (screen*)	229,321
S3	S1 AND S2	283
S2	TI (familial OR hereditary OR inheritable OR bilateral) OR AB (familial OR hereditary OR inheritable OR bilateral) OR SU (familial OR hereditary OR inheritable OR bilateral)	108,612
S1	(MH "Retinoblastoma") OR TI (“Retinoblastoma*” OR “Retinal Neuroblastoma*” OR “Retinal Glioma*” OR "Retinal Glioblastoma*") OR AB (“Retinoblastoma*” OR “Retinal Neuroblastoma*” OR “Retinal Glioma*” OR "Retinal Glioblastoma*") OR SU (“Retinoblastoma*” OR “Retinal Neuroblastoma*” OR “Retinal Glioma*” OR "Retinal Glioblastoma*")	1,515

Final search highlighted in yellow.

Table S4. Wiley/Cochrane Library search on Feb 12 2021 (3).

ID	Search	Hits
#1	(“Retinoblastoma*” OR (Retinal NEXT Neuroblastoma*) OR (Retinal NEXT Glioma*) OR (Retinal NEXT Glioblastoma*)):ti,ab,kw	137
#2	familial:ti,ab,kw OR hereditary:ti,ab,kw OR inheritable:ti,ab,kw OR bilateral:ti,ab,kw	23485
#3	#1 AND #2	13
#4	screen*:ti,ab,kw	74417
#5	#3 AND #4	0
#6	(early NEXT diagnos*):ti,ab,kw OR (early NEXT detect*):ti,ab,kw	5523
#7	#3 AND #6	0
#8	registry:ti,ab,kw OR registries:ti,ab,kw OR register:ti,ab,kw OR registers:ti,ab,kw	29602
#9	#3 AND #8	1
#10	(age NEAR/4 detection):ti,ab,kw OR (age NEAR/4 diagnosis):ti,ab,kw OR (age NEAR/4 onset):ti,ab,kw OR (age NEAR/4 presentation):ti,ab,kw	6923
#11	#3 AND #10	2
#12	#5 OR #7 OR #9 OR #11	3

Final search highlighted in yellow.
